# Regulation Effect of Zinc Fingers and Homeoboxes 2 on Alpha-Fetoprotein in Human Hepatocellular Carcinoma

**DOI:** 10.1155/2013/101083

**Published:** 2013-02-26

**Authors:** Shao Wei Hu, Meng Zhang, Ling Xue, Jian Ming Wen

**Affiliations:** ^1^Department of Pathology, The First Affiliated Hospital, Sun Yat-sen University, Guangzhou, China; ^2^Department of Pathology, Guangdong Hospital of Chinese Traditional Medicine, Guangzhou, China

## Abstract

*Aim*. To investigate the relationship between alpha-fetoprotein and zinc fingers and homeoboxes 2 in hepatocellular carcinoma. *Materials and Methods*. The expressions of zinc fingers and homeoboxes 2, nuclear factor-YA, and alpha-fetoprotein mRNA in 63 hepatocellular carcinoma were detected by reverse transcriptase-polymerase chain reaction and compared with the clinical parameters of the patients. Selectively, silence of zinc fingers and homeoboxes 2 in HepG2 cells was detected by RNA interference technique. *Results*. Alpha-fetoprotein mRNA expression was detected in 60.3% of hepatocellular carcinoma cases. Zinc fingers and homeoboxes 2 mRNA expression (36.5%) was significantly negatively correlated with serum alpha-fetoprotein concentration and mRNA expression. A strong positive correlation was found between zinc fingers and homeoboxes 2 and nuclear factor-YA mRNA expression (42.9%), while the latter was negatively correlated with serum alpha-fetoprotein concentration and mRNA expression. Treatment with zinc fingers and homeoboxes 2 small interfering RNA led to 85% and 83% silence of zinc fingers and homeoboxes 2 mRNA and protein expression and 60% and 61% reduction of nuclear factor-YA mRNA and protein levels in the HepG2 cells, respectively. Downregulation of zinc fingers and homeoboxes 2 also induced a 2.4-fold increase in both alpha-fetoprotein mRNA and protein levels. *Conclusions*. Zinc fingers and homeoboxes 2 can regulate alpha-fetoprotein expression via the interaction with nuclear factor-YA in human hepatocellular carcinoma and may be used as an adjuvant diagnostic marker for alpha-fetoprotein-negative hepatocellular carcinoma.

## 1. Introduction

Alpha-fetoprotein (AFP) is one of the major serum proteins in fetal mammals. Its concentration dramatically decreases after birth and remains at a low basal level in adults [1]. Olsson et al. found that the average AFP level in the BALB/cJ mice is about 10-fold higher than that of the controls (C3H/He and BALB/c/BOM) 9-10 weeks postnatally [2]. The postnatal AFP level in BALB/cJ mice is controlled by a single recessive Mendelian gene, previously named raf (regulation of alpha-fetoprotein) and renamed Afr1 (alpha-fetoprotein regulator 1) [3]. Afr1 governs postnatal AFP mRNA levels in adult mice liver [4, 5]. The AFP promoter is the target of Afr1-mediated postnatal repression [6]. Recently, Afr1 was identified as zinc fingers and homeoboxes 2 (ZHX2). In adult BALB/cJ mice, retrotransposon insertion in ZHX2 reduces its mRNA expression, resulting in an elevated expression of AFP [7].

Our previous studies have demonstrated reduced ZHX2 expression in hepatocellular carcinoma (HCC) [8]. This expression silence is involved in hypermethylation of the ZHX2 gene promoter [9]. Shen et al. [10] also have shown that the expression level of AFP in HepG2 cells is remarkably reduced by transfection of ZHX2 vector into the cells. In contrast, using siRNA inhibition technique AFP is derepressed in LO2 and SMMC7721 cells, when ZHX2 levels are reduced. ZHX2 repression is governed by the AFP promoter and requires intact nuclear factor (HNF1) binding sites.

Nuclear factor-Y (NF-Y) is an ubiquitous transcription factor that is an comprised of three subunits: NF-YA, NF-YB, and NF-YC [11]. The YB and YC subunits form a tightly bound dimer that presents a complex surface for the subsequent association of the YA subunit [12, 13]. The resulting trimer binds to an inverted CCAAT box, stimulating the transcription of a number of genes [14]. The NF-YA subunit contains two activation domains: a glutamine-rich region and a serine/threonine-rich region. ZHX2 residues 263–497 interact with the latter region. Immunoprecipitation analysis detected an interaction between ZHX2 and NY-FA in human embryonic kidney cells. Moreover, ZHX2 regulates NF-YA-regulable genes such as cdc25C [15]. Thus, ZHX2 can form homodimers or heterodimers with other ZHX members [16], then interacts with the activation domain of NF-YA, and represses transcription of its regulable gene [17]. 

In fact, AFP gene could be reactivated in human HCC [18]. The serum AFP levels were typically markedly elevated in HCC patients [19]. ZHX2 promoter hypermethylation could cause a low mRNA expression of ZHX2 in HCC [9]. However, the association between ZHX2, NF-YA, and AFP expressions in HCC has not been documented. It is also not clear whether ZHX2 regulates AFP gene expression by interacting with NF-YA in HCC. In this paper, we studied ZHX2, NF-YA, and AFP expressions in human HCC tissues by reverse transcriptase-polymerase chain reaction (RT-PCR). We also used RNA interference (RNAi) technology to selectively silence ZHX2 in HepG2 cells in order to clarify the possible regulation of AFP expression.

## 2. Materials and Methods

### 2.1. HCC Tissues

The study was approved by the Ethics Committee of The First Affiliated Hospital of Sun Yat-sen University, Guangzhou, China, and informed consent was obtained from the participating patients. Clinical samples were obtained by hepatectomy from 63 HCC patients (53 males, 10 females; age range 20–70 years; average age of 47 years) at the First Affiliated Hospital of Sun Yat-sen University. The collected cancer tissues were immediately frozen in liquid nitrogen and stored at −80°C for RT-PCR analysis. None of the cases received adjuvant therapy before operation.

The tumor grade was described by Edmondson and Steiner [20] and classified as grade I (2 cases), grade II (41 cases), grade III (18 cases), and grade IV (2 cases). Hepatitis B surface antigen (HBsAg) was positive in the serum of patients examined (56/56), in the rest 7 cases it was not done. The tumor size was less than 5 cm in 18 cases and larger than 5 cm in 45. Only 13 (20.7%) in all 63 cases had normal serum AFP concentration (<20 *μ*g/L). The cut-off for normal AFP level (20 *μ*g/L) and tumor size (5 cm) was according to previous studies [15, 18]. In addition, there were 28 cases with metastases, in the portal vein (17 cases), lymph node (4 cases), extrahepatic bile duct (2 cases), adrenal gland (2 cases), stomach (1 case), and peritoneal dissemination (2 cases). Cirrhosis was observed in 32 of 63 adjacent nontumorous tissues.

### 2.2. RNA Preparation and RT-PCR

Total RNA was extracted by using TRIzol Reagent (Invitrogen, Carlsbad, CA, USA) according to the manufacturer's guidelines. The concentration and purity of RNA were determined by measuring the absorbance at 260 and 280 nm. The RNA was dissolved in diethylpyrocarbonate-treated water to a final concentration of 1 *μ*g/*μ*L.

Total RNA (5 *μ*g) was reverse-transcribed into the first-strand cDNA at 42°C for 1 hour in 20 *μ*L reaction mixtures consisting of oligo(dT)_18_ primer (0.5 *μ*g), RiboLock Ribonuclease inhibitor (20 units), 10 mM dNTP mix (2 *μ*L), and RevertAid M-MuLV Reverse Transcriptase (200 units) (Fermentas Life Sciences, European Union). All cDNAs were then subjected to amplification with primers for ZHX2, AFP, NF-YA, and glyceraldehyde-3-phosphate dehydrogenase (GAPDH); the latter served as an internal standard. The primers spanned intron/exon boundaries (Table 1). Prior to the amplification of the experimental samples, the amount of cDNA in all of the samples was equalized. In addition, optimal conditions including Mg^2+^ concentration and annealing temperature for each set of primers were determined. Subsequently, optimization for the number of PCR cycles was determined for linear amplification. PCR was carried out in a Gene Amp PCR System 9600 (Perkin Elmer, Foster City, CA, USA). The PCR products were analyzed on an 8.0% acrylamide gel and the images of the silver nitrate stained bands were obtained with a Nikon E4500 digital camera (Nikon Corp., Tokyo, Japan). As a negative control, the cDNA template was omitted in the reaction. The RT-PCR was performed at least twice in independent experiments.

### 2.3. Cell Culture

Human hepatocellular carcinoma cells (HepG2) were cultured at 37°C in a humidified incubator with 5% CO_2_ and 95% air atmosphere in RPMI 1640 (Gibco BRL, Grand Island, NY, USA) supplemented with 10% fetal calf serum (FCS), 2 mmol/L L-glutamine, 100 *μ*g/mL streptomycin, and 100 IU/mL penicillin (Hyclone, Bio-Check Laboratories Ltd., USA).

### 2.4. Small Interfering RNA (siRNA) and Transfection

The sequences of human ZHX2 specific siRNA were 5′- GACACAUUAGGACACGUCAdAdA-3′ (sense) and 5′-dAdACUGUGUAAUCCUGUGCAGU-3′ (antisense). As a control siRNA, we used a corresponding nonsilencing siRNA with the sequences 5′-GACACAGAUACGAUCGUCAdAdA-3′ (sense) and 5′-dAdACUGUGUCUAUGCUAGCAGU-3′ (antisense). All synthetic RNA oligonucleotides were synthesized and purified at Ribobio (Guangzhou, China).

One day before transfection, HepG2 cells were seeded at a density of 3 × 10^5^ cells/well in a complete medium without antibiotics in 12-well plates. The siRNA (either ZHX2 siRNA or control siRNA) was diluted in a final volume of 100 *μ*L of serum-free Opti-MEM (Gibco) medium. In a separate tube, 2 *μ*L Lipofectamine 2000 (Invitrogen, Carlsbad CA, USA) was mixed into a final volume of 100 *μ*L of serum-free Opti-MEM medium per well and incubated for 5 minutes at room temperature. The two mixtures were mixed gently and incubated for another 20 minutes. Add the complexes to the well containing cells and medium. The final concentration of siRNA was 50 nM. Cells were subsequently cultured for 48 hours before further analysis (RT-PCR and Western blot).

### 2.5. Protein Preparation and Western Blot

HepG2 cells were treated for 30 minutes on ice with lysis buffer (RIPA, Shenerg Biocolor, China). Protein concentrations were determined using the bicinchoninic acid protein assay. Equal amounts of cellular protein were separated by 10% sodium dodecyl sulfate-polyacrylamide gel electrophoresis (SDS-PAGE) and transferred to polyvinylidene difluoride membranes. The membranes were incubated in blocking buffer consisting of Tris-buffered saline (TBS) containing 5% nonfat dry milk for 1 hour at room temperature. The membranes were then incubated with the primary antibodies against ZHX2 (1 : 4000 dilution, ABNOVA Corporation, Taiwan), NF-YA (1 : 200 dilution, Santa Cruz, CA, USA), AFP (1 : 200 dilution, NeoMarkers, Fremont, CA, USA), and beta-actin (1 : 200 dilution, Boster, China). Horseradish peroxidase-labeled anti-mouse secondary antibody (1 : 1000 dilution, DAKO, Carpinteria, CA, USA) was applied onto the blots. After incubation with the electrochemiluminescence (ECL, Applygen Technologies Inc.), reagent, immunochemiluminescence signals were recorded on X-ray film.

### 2.6. Statistical Analysis

Statistical differences were evaluated using *χ* ^2^ test performed with SPSS11.5 for Windows software. A *P* value less than .05 for each test was considered statistically significant.

## 3. Results

**Figure 1:**
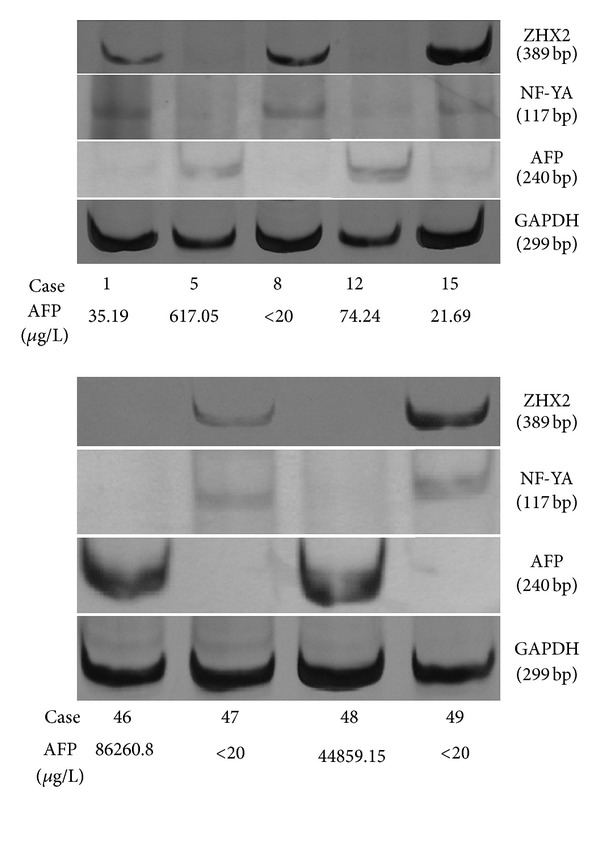
expression of zinc fingers and homeoboxes 2 (ZHX2), nuclear factor-YA (NF-YA), and alpha-fetal protein (AFP) mRNA in hepatocellular carcinoma (HCC) tissues. The target mRNA expression was detected by RT-PCR analysis. The expression of glyceraldehyde-3-phosphate dehydrogenase (GAPDH) served as an internal control. In cases 1, 8, 15, 47, and 49, ZHX2 and NF-YA mRNAs were detected, but AFP mRNA expression was not observed. In cases 5, 12, 46, and 48, ZHX2 and NF-YA mRNAs were not detected, but AFP mRNA expression was observed.

ZHX2 mRNA expression was detected in 23 (36.5%) of 63 HCC tissues (Figure 1). ZHX2 expression rate (69.2%) with less than 20 *μ*g/L AFP concentration in serum was significantly higher than that (28%) with more than 20 *μ*g/L serum AFP concentration (*P* = .009, OR = .17). Statistically, ZHX2 expression was not significantly associated with age, tumor size, cirrhosis, grading, and metastasis (Table 2).

NF-YA mRNA expression was detected in 27 (42.9%) of 63 HCC tissues (Figure 1). It was significantly negatively correlated with serum AFP concentration (*P* = .005, OR = .16). NF-YA expression was not significantly associated statistically with age, tumor size, cirrhosis, grading, and metastasis (Table 3).

AFP mRNA expression was detected in 38 (60.3%) of 63 HCC tissues (Figure 1). It was not significantly associated with age, tumor size, cirrhosis, grading, and metastasis. However, there was a significant association between AFP mRNA expression and serum AFP concentration (*P* = .001, OR = 14.14) (Table 4).

ZHX2 mRNA expression rate (26.3%) in HCC tissues with AFP mRNA expression was significantly lower than that (52%) without AFP mRNA expression (*P* = .04, OR = .33). In 27 HCC tissues with NF-YA expression, ZHX2 mRNA expression rate was 74.1%. In 36 NF-YA negative HCC tissues, only 3 ZHX2-positive tissues (8.3%) were detected. The difference was statistically significant (*P* = .001, OR = 31.43) (Table 5). Furthermore, NF-YA expression rate (31.6%) in HCC tissues with AFP expression was significantly lower than that (60%) without AFP expression (*P* = .03, OR = .31) (Table 6).

**Figure 2:**
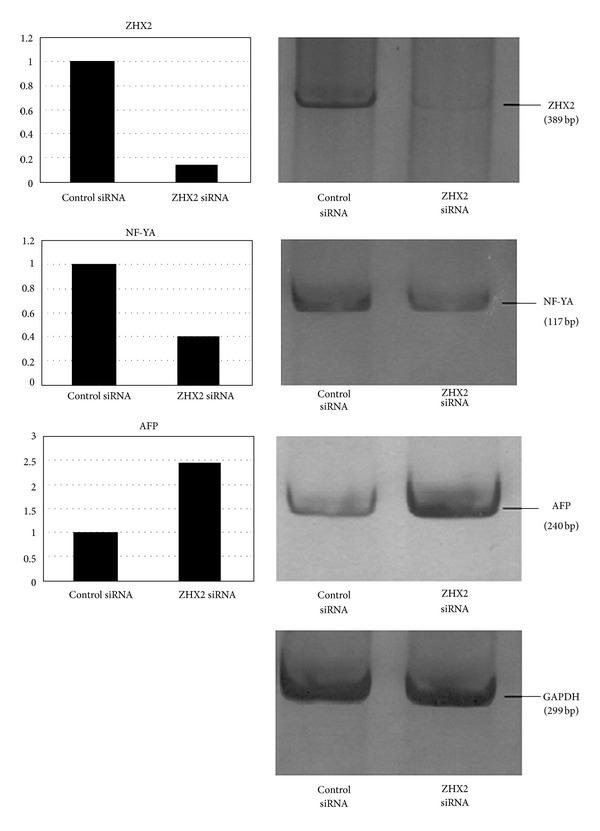
expression of zinc fingers and homeoboxes 2 (ZHX2), nuclear factor-YA (NF-YA), and alpha-fetal protein (AFP) mRNA in HepG2 cells after siRNA transfection. The target mRNA expression was detected by RT-PCR analysis. The expression of glyceraldehyde-3-phosphate dehydrogenase (GAPDH) served as an internal control. The amounts of mRNA in the ZHX2 siRNA-treated cells were compared to those of control cells. After siRNA transfection, ZHX2 mRNA expression (389 bp) was decreased by 85%, NF-YA mRNA expression (117 bp) was reduced by 60%, and AFP mRNA expression (240 bp) was increased by 2.4-fold.

**Figure 3:**
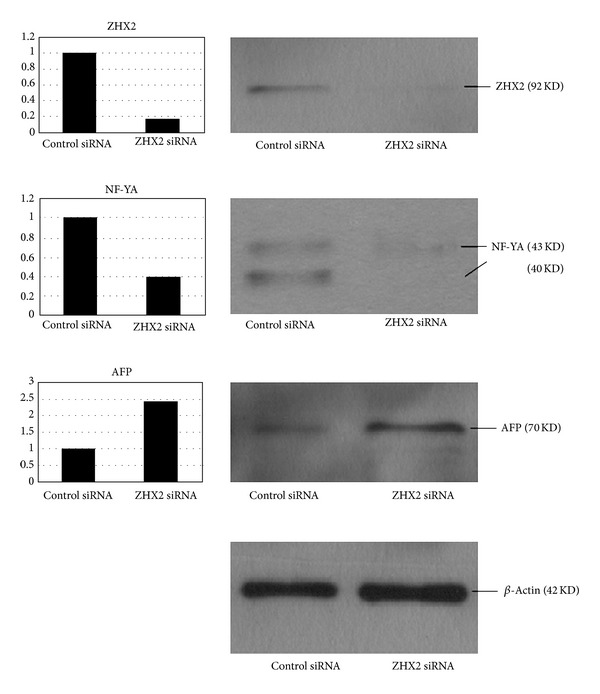
Expression of zinc fingers and homeoboxes 2 (ZHX2), nuclear factor-YA (NF-YA), and alpha-fetal protein (AFP) in HepG2 cells after siRNA transfection. The target protein expression was detected by Western blot analysis. The expression of *β*-actin was analyzed as an internal control. The amounts of protein in the ZHX2 siRNA-treated cells were compared to those of control cells. After siRNA transfection, ZHX2 protein expression (92** **KD) was decreased by 83%, NF-YA protein expression (40 KD and 43 KD) was reduced by 61%, and AFP protein expression (70 KD) was increased by 2.4-fold.

To further confirm the relations between ZHX2, NF-YA, and AFP, we used siRNA to silence the expression of ZHX2 and then detected the change of NF-YA and AFP expression in HepG2 cells. After transfection of siRNA into the cells, the level of ZHX2 mRNA and protein expression decreased significantly by 85% and 83%, respectively, as compared to control siRNA. Treatment with ZHX2 siRNA simultaneously led to 60% and 61% reduction in the NF-YA mRNA and protein levels in the cells, respectively. Downregulation of ZHX2 also induced a 2.4-fold increase in both AFP mRNA and protein levels (Figures 2 and 3).

## 4. Discussion

ZHX2 is a novel transcriptional repressor, which consists of 837 amino acid residues. The protein has two Cys2-His2-type zinc finger motifs and five homeodomains and is localized in the nuclei [15]. We previously found that promoter hypermethylation caused a low mRNA expression of ZHX2 in HCC. In this study, ZHX2 mRNA expression rate in HCC tissues was 36.5% and similar to the previous study (34.4%) [9]. Compared with clinicopathological parameters, ZHX2 mRNA expression was negatively associated with preoperative AFP level in serum. We also detected AFP mRNA expression in HCC tissues by RT-PCR, which was found to be lower in HCC tissues with ZHX2 expression. Taken together, these findings confirm a negative correlation between ZHX2 and AFP expressions in HCC. To investigate the regulation of AFP, we used RNAi technique because it is known to downregulate specific gene at a posttranscriptional level [21]. We found that the transfection of siRNA into HepG2 cells caused a silence of ZHX2 and an increased expression of AFP mRNA and protein. Yamada et al. also demonstrated that the promoter activity of alpha-fetoprotein was repressed by the expression of ZHX2 in HLE hepatoma cells in a dose-dependent manner [22]. They concluded that ZHX2 and ZHX3 were involved in the transcriptional repression of the HCC markers in normal hepatocytes, suggesting that the failure of the ZHX2 and/or ZHX3 expression might be a critical factor in hepatocyte carcinogenesis [22]. 

The interaction of ZHX2 with the serine/threonine-rich AD of NF-YA has been previously confirmed [15]. By RT-PCR analysis, we found that ZHX2 expression was positively correlated with NF-YA expression. Treatment with ZHX2 siRNA led to 60% and 61% reduction in the NF-YA mRNA and protein levels, respectively. These results indicated that ZHX2 was correlated to NF-YA in HCC. It is possible that ZHX2 downregulates gene expression via the interaction with NF-YA in HCC.

Furthermore, we analyzed NF-YA and AFP expressions in HCC tissues and found that NF-YA expression was negatively correlated to AFP expression and serum AFP level. Treatment with ZHX2 siRNA could lead to decreased NF-YA and increased AFP at the mRNA and protein levels. Thus, it is possible that ZHX2 regulates AFP transcription via the interaction with NF-YA in HCC. Although there is no evidence to show that NF-YA directly interacts with AFP, NF-YA might regulate AFP expression indirectly through other genes, such as p300 [14] and p53 [23]. Further studies are required to explain the exact mechanism(s) of AFP regulation in HCC.

In conclusion, we detected ZHX2, NF-YA, and AFP expressions in human HCC tissues by RT-PCR and found a close correlation between ZHX2 and NF-YA and a negative relation between ZHX2 and AFP. The RNAi of ZHX2 in HepG2 cells further verified these relations. Therefore, AFP can be regulated by ZHX2 in HCC, and this regulation may be via the interaction with NF-YA. ZHX2 may be used as an adjuvant diagnostic tissue marker for AFP-negative HCC.

 Further study is required to detect the expression levels of ZHX2 and AFP in hepatic cirrhosis and dysplastic nodules, in order to understand the expression difference in both lesions and establish the possibility of ZHX2 as an earlier screening marker for patients with cirrhosis and/or preneoplastic nodule.

## Figures and Tables

**Table 1:** Primer sequences for RT-PCR analysis.

Gene	Primer sequences (5′-3′)	Annealing temperature (°C)	Cycle numbers	Amplified products (bp)
ZHX2	Sense: GGTAGCGACGAGAACGAG Antisense: AGGACTTTGGCACTATGAAC	58	34	389
NF-YA	Sense: GAGTCTCGGCACCGTCAT Antisense: TGCTTCTTCATCGGCTTG	57	34	117
AFP	Sense: GTTGCCAACTCAGTGAGGAC Antisense: GAGCTTGGCACAGATCCTTA	59	28	240
GAPDH	Sense: GCTGAGAACGGGAAGCTTGT Antisense: GCCAGGGGTGCTAAGCAGTT	58	30	299

AFP: alpha-fetoprotein, bp: base pair, GAPDH: glyceraldehyde-3-phosphate dehydrogenase, NF-Y: nuclear factor-Y, and ZHX2: zinc fingers and homeoboxes 2.

**Table 2:** Relationship between ZHX2 mRNA expression and clinicopathological parameters in HCC tissues.

Parameter	+ (%)	ZHX2 mRNA *P* value	OR (95% CI)
Total	23 (36.5)		
AFP (*μ*g/L)			
<20	9 (69.2)	.009	.17 (.05–.65)
>20	14 (28)		
Tumor size (cm)			
≤5	7 (38.9)	.80	.87 (.28–2.68)
>5	16 (35.6)		
Background liver			
Without cirrhosis	12 (38.7)	.72	.83 (.30–2.32)
With cirrhosis	11 (34.4)		
Grade			
I-II	15 (34.9)	.70	1.24 (.42–3.71)
III-IV	8 (40)		
Metastasis			
Without	15 (42.9)	.24	.53 (.185–1.54)
With	8 (28.6)		

CI: confidence interval, HCC: hepatocellular carcinoma, OR: odds ratio, and ZHX2: zinc fingers and homeoboxes 2.

**Table 3:** Relationship between NF-YA mRNA expression and clinicopathological parameters in HCC tissues.

Parameter	+ (%)	NF-YA mRNA *P* value	OR (95% CI)
Total	27 (42.9)		
AFP (*μ*g/L)			
<20	10 (76.9)	.005	.16 (.04–.64)
>20	17 (34)		
Tumor size (cm)			
≤5	7 (38.9)	.69	1.26 (.41–3.84)
>5	20 (44.4)		
Background liver			
Without cirrhosis	15 (48.4)	.38	.64 (.23–1.75)
With cirrhosis	12 (37.5)		
Grade			
I-II	16 (37.2)	.18	2.06 (.70–6.05)
III-IV	11 (55)		
Metastasis			
Without	18 (51.4)	.27	.56 (.20–1.57)
With	9 (32.1)		

CI: confidence interval, HCC: hepatocellular carcinoma, NF-Y: nuclear factor-Y, and OR: odds ratio.

**Table 4:** Relationship between AFP mRNA expression and clinicopathological parameters in HCC tissues.

Parameter	+ (%)	AFP mRNA *P* value	OR (95% CI)
Total	38 (60.3)		
AFP (*μ*g/L)			
<20	2 (15.4)	.001	14.14 (2.78–72.05)
>20	36 (72)		
Tumor size (cm)			
≤5	9 (50)	.29	1.81 (.60–5.49)
>5	29 (64.4)		
Background liver			
Without cirrhosis	22 (71)	.09	.41 (.15–1.16)
With cirrhosis	16 (50)		
Grade			
I-II	29 (67.4)	.09	.40 (.13–1.17)
III-IV	9 (45)		
Metastasis			
Without	21 (60)	.95	1.03 (.37–2.85)
With	17 (60.7)		

AFP: alpha-fetoprotein, CI: confidence interval, HCC: hepatocellular carcinoma, and OR: odds ratio.

**Table 5:** Relationship between ZHX2, NF-YA, and AFP mRNA expressions in HCC tissues.

	+ (%)	ZHX2 mRNA *P* value	OR (95% CI)
NF-YA mRNA			
−	3 (8.3)	.001	31.43 (7.28–135.62)
+	20 (74.1)		
AFP mRNA			
−	13 (52)	.04	.33 (.11–.96)
+	10 (26.3)		

AFP: alpha-fetoprotein, CI: confidence interval, HCC: hepatocellular carcinoma, NF-Y: nuclear factor-Y, OR: odds ratio, and ZHX2: zinc fingers and homeoboxes 2.

**Table 6:** Relationship between  NF-YA and AFP mRNA expression in HCC tissues.

	+ (%)	NF-YA mRNA *P* value	OR (95% CI)
AFP mRNA			
−	15 (60)	.03	.38 (.11–.88)
+	12 (31.6)		

AFP: alpha-fetoprotein, CI: confidence interval, HCC: hepatocellular carcinoma, NF-Y: nuclear factor-Y, and OR: odds ratio.
